# [^18^F]FDG Positron Emission Tomography within Two Weeks of Starting Erlotinib Therapy Can Predict Response in Non-Small Cell Lung Cancer Patients

**DOI:** 10.1371/journal.pone.0087629

**Published:** 2014-02-05

**Authors:** Mammar Hachemi, Olivier Couturier, Laurent Vervueren, Pacôme Fosse, Franck Lacœuille, Thierry Urban, José Hureaux

**Affiliations:** 1 LUNAM Université, 49 000, Angers, France; 2 Université d'Angers, CHU Angers, Pôle de Radiologie, Service de Médecine Nucléaire, Angers, France; 3 INSERM UMR_S 1066 Micro et Nanomédecines Biomimétiques, Angers, France; 4 Université d'Angers, CHU Angers, Pôle des Spécialités Médicales et Chirurgicales Intégrées, Département de Pneumologie, Angers, France; 5 Université d'Angers, Equipe Pyver, Angers, France; University of Nebraska Medical Center, United States of America

## Abstract

**Purpose:**

The aim of this prospective study was to evaluate whether [^18^F]FDG-PET/CT, performed within two weeks of starting erlotinib therapy can predict tumor response defined by RECIST 1.1 criteria after 8 weeks of treatment in patients with inoperable (stage IIIA to IV) non-small cell lung cancer patients.

**Patients and Methods:**

Three [^18^F]FDG-PET/CT scans were acquired in 12 patients before (5±4 days) and after 9±3 days (early PET) and 60±6 days (late PET) of erlotinib therapy. Conventional evaluation, including at least chest CT (baseline versus after 8 weeks of treatment), was performed according to RECIST 1.1 criteria. Change in [^18^F]FDG uptake was compared with conventional response, progression-free survival (PFS), and overall survival (OS).

**Results:**

By using ROC analysis, the Area Under the Curve for prediction of metabolic non-progressive disease (mNP) by early PET was 0.86 (95% CI, 0.62 to 1.1; P = 0.04) at a cut-off of 21.6% reduction in maximum Standardized Uptake Value (SUVmax). This correctly classified 11/12 patients (7 with true progressive disease; 4 with true non-progressive disease; 1 with false progressive disease). Non-progressive disease after 8 weeks of treatment according to RECIST 1.1 criteria was significantly more frequent in patients classified mNP (P = 0.01, Fisher's exact test). mNP patients showed prolonged PFS (HR = 0.27; 95% CI, 0.04 to 0.59; P<0.01) and OS (HR = 0.34; 95% CI, 0.06 to 0.84; P = 0.03). Late PET analysis provided concordant results.

**Conclusion:**

Morphologic response, PFS and OS survival in non-small cell lung cancer patients can be predicted by [^18^F]FDG-PET/CT scan within 2 weeks after starting erlotinib therapy.

## Introduction

Lung cancer is the leading cause of cancer-related death in both Europe[1] and the United States of America.[2] The most common forms of lung cancer are non-small cell lung cancer (NSCLC) histological subtypes. Systemic chemotherapy has contributed to a significant improvement in NSCLC therapy, but progress appears to be stagnating.[3], [4] Over the last decade, a better knowledge of cellular pathways has allowed the development of new therapies based on NSCLC-driving genetic abnormalities. Targeted therapies have been developed to block pathological cellular pathways involved in cancer cell survival, proliferation and metastasis. Epidermal Growth Factor Receptor (EGFR) is overexpressed in NSCLC[5] and has been extensively studied as a potential therapeutic target. Two EGF Receptor blockers, gefitinib and erlotinib, have been demonstrated to be effective in front-line therapy in patients with inoperable NSCLC harboring EGFR-activating mutations.[6], [7] Erlotinib is also authorized after failure of previous chemotherapy and as maintenance therapy.[8], [9]

In clinical practice, evaluation of tumor response is based on changes in tumor size, according to criteria proposed by the World Health Organization[10] or RECIST criteria.[11], [12] This morphological evaluation may lead to underestimation of the efficacy of cytostatic therapeutic agents such as erlotinib that stabilize the disease in non-mutated patients, whereas conventional cytotoxic drugs induce shrinkage of tumor dimensions in the case of tumor response. NSCLC tumor size evaluation can also be difficult due to atelectasis of normal lung. The major limitations to morphological imaging methods are their inability to assess response to therapy at an early stage and their inability to identify cancer in residual masses after treatment.

In patients with NSCLC, [^18^F]FDG-PET has been recognized as an adequate staging tool[13], [14] and several studies also suggest that the standardized uptake value (SUV) has a prognostic value in NSCLC.[15], [16] The value of SUV for evaluation of tumor response to targeted therapy is currently being investigated. We designed a preliminary study to evaluate tumor response in NSCLC patients eligible for erlotinib therapy. The aim of this prospective study was to determine whether [^18^F]FDG-PET/CT, performed several days after starting erlotinib therapy, could predict tumor response defined by RECIST 1.1 criteria and [^18^F]FDG-PET/CT after 8 weeks of treatment.

## Materials and Methods

### Patients

Twelve consecutive eligible patients with stage IIIA to IV NSCLC (7 adenocarcinomas, 3 large cell carcinomas, 2 squamous cell carcinomas), in whom erlotinib therapy was indicated, were studied at the Angers University Hospital, France. Screening for EGF receptor mutations was carried out (patient characteristics are shown in Table 1). Eligibility criteria were: histologically or cytologically proven NSCLC; unresectable stage III/IV disease or recurrent disease after surgery; age over 18 years; measurable disease according to RECIST 1.1 criteria; Eastern Cooperative Oncology Group (ECOG) performance status between 0 to 2; adequate bone marrow function, liver function, and renal function. Patients were not included if they had previous lung diseases such as interstitial pneumonitis or lung fibrosis identified by chest Computed Tomography (CT) scan or diabetes mellitus that could artefact PET imaging. Life expectancy was predicted to be longer than 12 weeks. Erlotinib was administered orally in a dosage of 150 mg/day on an empty stomach until clinical disease progression, unacceptable toxicity or patient refusal. The medical ethics committee of the CHU of Angers approved the study protocol. All patients gave informed written consent before inclusion according to local medical ethical committee regulations and in accordance with the guidelines established by the World Medical Association Declaration of Helsinki.

**Table 1:** Clinical characteristics of the study population.

Patients	
Male	6 *(50)*
Female	6 *(50)*
Total	12 *(100)*
Histology	
Adenocarcinoma	7 *(58)*
Large cell carcinoma	3 *(25)*
Squamous cell carcinoma	2 *(17)*
Clinical stage	
IIIA or IIIB	2 *(17)*
IV	10 *(83)*
Smoking status	
Current	5 *(42)*
Former	2 *(17)*
Never	5 *(42)*
EGFR mutation status	
Presence	2 *(17)*
Absence	10 *(83)*
Previous chemotherapy	
Yes	10 *(83)*
No	2 *(17)*
Size of primary tumor (cm)	
1.0–2.0	4 *(33)*
2.1–3.0	3 *(25)*
3.1–5.0	5 *(42)*
>5.1	1 *(8)*
Metastasis	
Lymph nodes	12 *(100)*
Lung	4 *(33)*
Liver	2 *(17)*
Bone	4 *(33)*
Adrenal glands	0

### Work Plan (study design)

#### [^18^F]FDG PET/CT imaging

Three [^18^F]FDG PET/CT scans were planned: PET1 before starting therapy, PET2 within 2 weeks after starting therapy and a third [^18^F]FDG PET/CT scan (PET3) 8 weeks after starting erlotinib therapy.

PET/CT examinations were obtained in 2D mode from the vertex to mid-thighs (5 minutes of emission scan per bed position with an average of 7 bed positions at 15 cm intervals) (Discovery-ST, GE Healthcare, France). Patients were instructed to fast for at least 6 hours prior to scanning.

Unenhanced CT scan was performed from the skull base to the upper thighs. CT parameters were 120 kVp, 100 mAs, 0.8 second rotation, 3.27 mm slice collimation, and Pitch 1.5.

CT data were used for attenuation correction, and PET images were reconstructed by clinical standard 2D-iterative algorithm (ordered subset expectation maximization using 4 iterations and 16 subsets; zoom 100%; image matrix size: 128×128; and Gaussian post-smoothing of 5 mm in full width at half maximum).

No corrections for partial volume effect, lean body mass, or blood glucose levels were applied.

#### Conventional evaluation

Conventional staging and follow-up were performed according to standards of care.[11], [12] Conventional evaluation included at least clinical examination plus CT scan performed before (CT1; 7±6 days) and 8 weeks after (CT2; 58±8 days) starting erlotinib therapy. None of the patients underwent additional CT scanning during the 2 weeks after starting erlotinib therapy.

Chest, abdomen and pelvis CT scans (Brillance 64 PHILIPS Medical System®, France) were acquired from the lung apex to the symphysis pubis after an intravenous embolus of 130 mL of iodinated contrast agent (Xenetix350^®^). Helical scanning parameters were 130 kVp, 120 mAs, 1 second rotation, 4 mm slice collimation, 8 mm/s bed speed and 3 mm section width.

### Image analysis and response evaluation

CT data were interpreted by two experienced physicians specialists in thoracic oncology blinded to PET/CT results according to the Response Evaluation Criteria in Solid Tumors (RECIST 1.1 criteria[12]) by comparison of baseline CT scan (CT1) and final CT scan (CT2). Therapeutic response evaluation was defined as: 1) complete response (CR: disappearance of all target lesions); 2) partial response (PR: at least 30% decrease in the sum of the longest diameter of five target lesions); 3) progressive disease (PD: at least a 20% increase in the sum of the longest diameter of five target lesions); and 4) stable disease (SD: neither sufficient shrinkage to qualify for PR nor sufficient increase to qualify for PD). Patients were then classified in the progressive disease (P) group or the non-progressive disease (NP) group, including CR, PR and SD therapeutic response.

[^18^F]FDG PET interpretation was performed on an Imagys^®^ workstation (Keosys, Saint-Herblain, France), qualitatively and semi-quantitatively by two experienced nuclear medicine physicians, blinded to clinical and conventional evaluation results. Any focus of increased [^18^F]FDG uptake over background not located in areas of normal [^18^F]FDG uptake and/or [^18^F]FDG excretion was considered to be positive for tumor. For semi-quantitative analyses of [^18^F]FDG uptake, 3D regions of interest (VOIs) were placed over all lesions considered to be positive for tumor by using Imagys^®^ software (Keosys, France). The maximum standardized uptake value (SUVmax) was calculated using the single hottest pixel inside the tumor VOI. SUV peak was also calculated using a 1.2 cm diameter spherical VOI containing the SUVmax. Bone lesions were not taken into account, as they were considered to be non-measurable lesions.

For patients with more than one tumor lesion, the sum of SUVmax and SUVpeak were calculated and used for evaluation of changes between PET1 and PET2. PET measurements were performed in up to a maximum of five measurable target lesions.

All SUVs were normalized to the injected dose and patient body weight. The percentage changes in SUV between PET1 and PET2 were finally calculated as follows: Δ_SUV_ = (SUV_1_−SUV_2_)/SUV_1_. The same protocol was used for PET1 and PET3.

### Statistical analysis

Data are expressed as mean±SD, excepted for survival data that were expressed as the median. The primary endpoint of the study was comparison of changes in tumor [^18^F]FDG uptake on PET2 versus PET1, PET3 versus PET1 and subsequent CT scan evaluation at 8 weeks after initiation of erlotinib therapy. Friedman test was used for non-parametric comparison of repeated measures. The secondary endpoints were to determine the Receiver Operating Characteristic (ROC) analysis for [^18^F]FDG changes with regard to predicting response to erlotinib therapy. The relationship between metabolic response (patients stratified according to the median value of SUV variations) and clinical response was analyzed by Fisher's exact test. Progression-free survival (PFS) and overall survival (OS) were determined by standard Kaplan-Meier survival analysis, and between-group comparison was performed by log-rank test. PFS was defined as the time interval from the date of enrolment in the study until the first signs of progression. OS was calculated from the date of enrolment until death from any cause. All analyses were performed using Graphpad prism version 4.0 b 2004 (Graphpad Software, San Diego, CA). The limit of significance was set at 0.05.

## Results

### Population

Twelve eligible patients with NSCLC, 6 women (50%) and 6 men (50%) with a mean age of 60±13 years, were included. Two patients presented tumors harboring an activating Epidermal Growth Factor Receptor mutation (2573T>G substitution (p.Leu858Arg) in exon 21 in one patient; deletion (L747_E749del) in exon 19 in the other patient). Patient characteristics are described in Table 1. The median duration of erlotinib therapy was 75 days. Due to rapid progression and death, PET3 and CT3 could not be performed in 2 patients.

### Tumor ^18^F-FDG uptake

The three [^18^F]FDG PET/CT scans were acquired as follows: PET1 5±4 days before starting therapy, PET2 9±3 days after starting therapy and PET3 60±6 days after starting erlotinib therapy. Scanning started 68±17 min (PET1), 71±16 min (PET2) and 64±13 min (PET3) after [^18^F]FDG injection of 271±53 MBq (PET1), 270±61 MBq (PET2) and 263±54 MBq (PET3). Blood glucose level was less than 1.5 g/L for all PET examinations, i.e. 1.1±0.1 g/L for PET1, 1.1±0.2 g/L for PET 2 and 1.1±0.2 g/L for PET3. Non-parametric Friedman tests did not show any significant difference between PET1, PET2, and PET3 for FDG uptake time, injected FDG dose or blood glucose.

Fifty-five lesions were described on PET1 before treatment and 45 lesions were defined as target lesions for PET evaluation of response to treatment (up to five most hypermetabolic lesions per patient; mean 3.8 lesions/patient). The mean tumor SUVmax of the most [^18^F]FDG–avid lesion (SUVmax) was 10.0±4.7 for PET1, and did not vary significantly over time with a mean of 10.1±6.6 for PET2 and a mean of 9.1±5.6 for PET3 (P = 0.97). The SUVpeak was 8.6±4.3 for PET1, 8.1±5.4 for PET2, and 7.1±4.6 for PET3 and did not vary over time (P = 0.60).

No variation over time was observed for the sums of SUV. The mean sum of tumor SUVmax of all target lesions was 30.1±19.5 for PET1, 27.5±17.7 for PET2, and 28.3±22.4 for PET3 (P = 0.83). Sums of SUVpeak of all target lesions were 22.7±14.3 for PET1, 20.6±13.4 for PET2, and 22.2±18.6 for PET3 (P = 0.44).

### [^18^F]FDG-PET response versus conventional evaluation

CT scan data were interpreted by chest physicians blinded to PET/CT scan results (Table 2). Evaluation of response to treatment according to RECIST 1.1 criteria demonstrated 7 patients with progressive disease (group P) and 5 patients with non-progressive disease (group NP) including 4 cases of stable disease (SD) and 1 partial response (PR).

**Table 2:** CT and PET assessments of response rates, OS and PFS.

Patient	PET2 versus PET1		PET3 versus PET1		RECIST 1.1 Evaluation		PFS	OS	New lesion
	*Δ SUV_max_* *	*Δ SUV_peak_* *	*Δ SUV_max_* *	*Δ SUV_peak_* *	*Response to Treatment*	*Progressive (P) or not (NP)*	*days*	*days*	*on PET3*
#1	−21.6	−17.6	18.6	−1.5	SD	NP	267	915	−
#2	25.9	26.9	70.3	77.4	PD	P	57	316	+
#3	9.0	7.6	23.4	23.3	PD	P	216	447	+
#4	−18.6	−15.0	−3.2	−2.6	PD	P	67	414	+
#5	−20.3	−11.1	42.1	51.1	PD	P	53	152	+
#6	−56.7	−59.9	−72.1	−70.6	PR	NP	190	296	−
#7	−22.0	−26.0	−31.3	−24.3	SD	NP	727	1249	+
#8	−32.0	−25.1	3.9	−3.9	SD	NP	317	1146	−
#9	16.4	7.8	−5.4	−10.8	SD	NP	77	359	−
#10	2.1	4.4	MD	MD	PD	P	37	92	MD
#11	36.1	20.0	30.3	25.7	PD	P	104	734	−
#12	−7.2	−10.5	MD	MD	PD	P	61	71	MD

* For patient with more than one tumor lesion, the sum of SUV_max_ and of SUV_peak_ were calculated and used for the evaluation of changes between PET1 and PET2 (or between PET1 and PET3). Missing data are indicated as MD.

**Figure 1:**
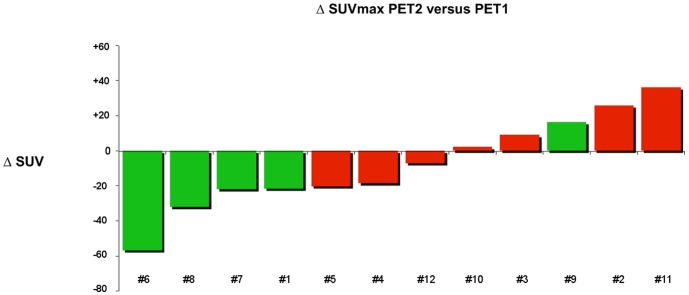
Percentage change in SUVmax on ^18^F-FDG PET/CT (cut-off: −21.6%) within 2 weeks of starting erlotinib therapy in relation to conventional imaging response. Each red or green bar represents a patient NP or P, respectively.

**Figure 2:**
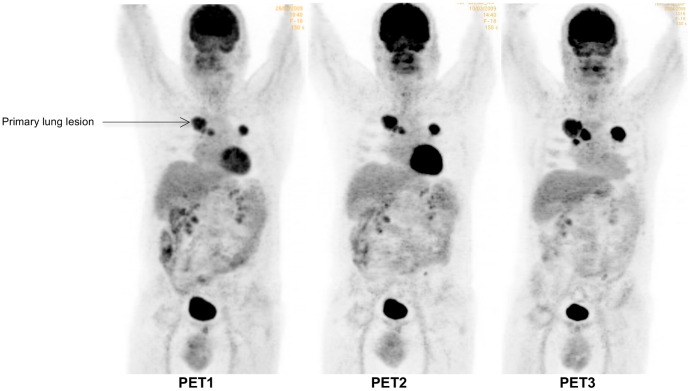
Example of a progressive patient on PET (mP) and conventional imaging. Progressive patient with right upper lobe NSCLC associated with médiastinal lymphadenopathy, lung and bone metastases (patient #2). Sum of the SUVmax of the 5 most hypermetabolic lesions (2 lung lesions, 2 mediastinal lymph nodes, one hilar lesion) were 35.2, 44.3 (+26%) and 59.9 (+70%) for PET1, PET2 (% versus PET1) and PET3 (% versus PET1), respectively. Based on a SUVmax cut-off value of −21.6, the patient was classified as mP on PET2, in accordance with RECIST evaluation on CT scan (performed 57 days after starting erlotinib). mP was confirmed on PET3 with the appearance of a new lesion (subcarinal adenopathy) and a 70% increase of SUVmax.

**Figure 3:**
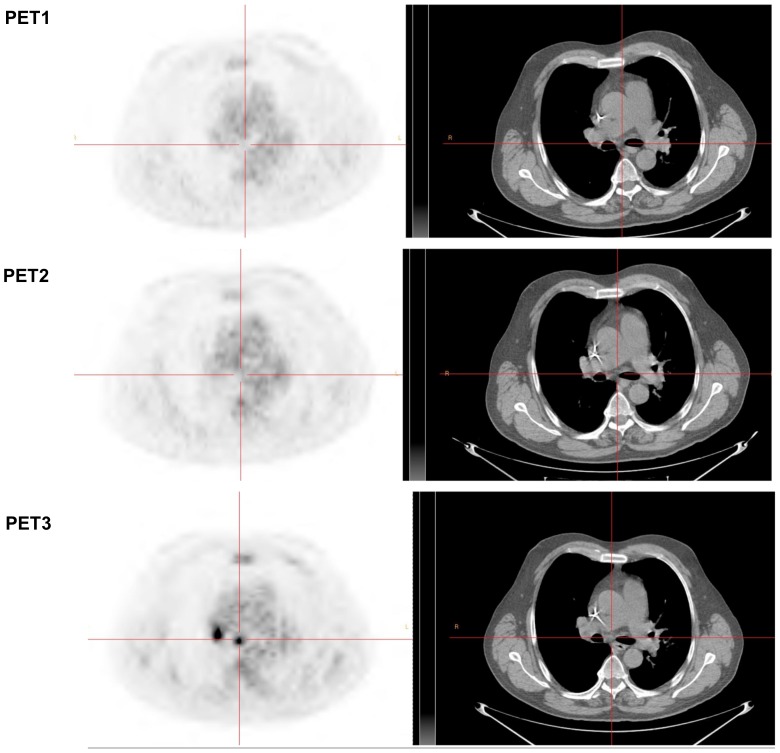
New subcarinal adenopathy on PET3 (same patient as Figure 2).

**Figure 4:**
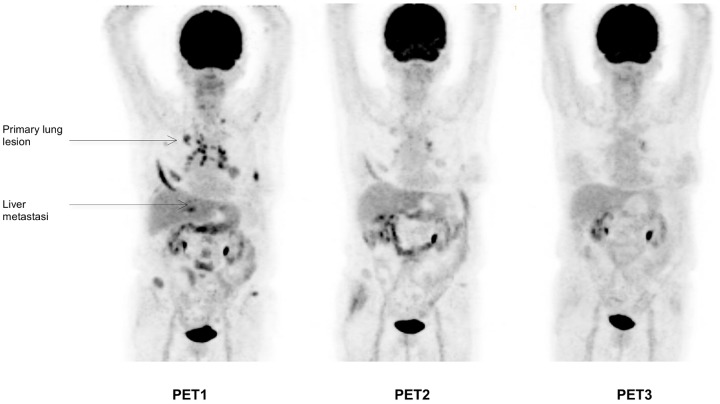
Example of an mNP patient. Non-progressive patient with right upper lobe NSCLC associated with mediastinal lymphadenopathy, lung, liver and bone metastases (patient #6). Sum of the SUVmax of the 5 most hypermetabolic lesions (2 lung lesions, 2 mediastinal lymph nodes, one liver lesion) were 45.6, 19.7 (−56.7%) and 12.7 (−72%) for PET1, PET2 (% versus PET1) and PET3 (% versus PET1), respectively. Based on a SUVmax cut-off value of −21.6, the patient was classified as mNP on PET2, in accordance with RECIST evaluation on CT scan (performed 58 days after starting erlotinib). mNP was confirmed on PET3 with almost complete extinction of the various lesions and a 72% decrease of SUVmax.

**Figure 5:**
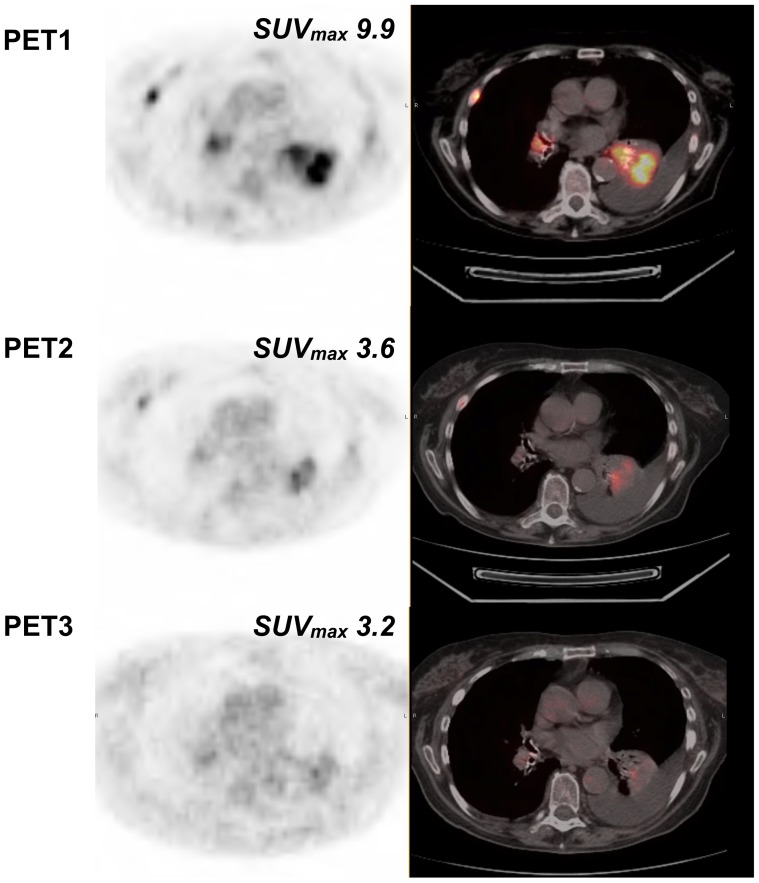
Example of left lower lobe pulmonary target lesion (same patient as Figure 4).

**Figure 6:**
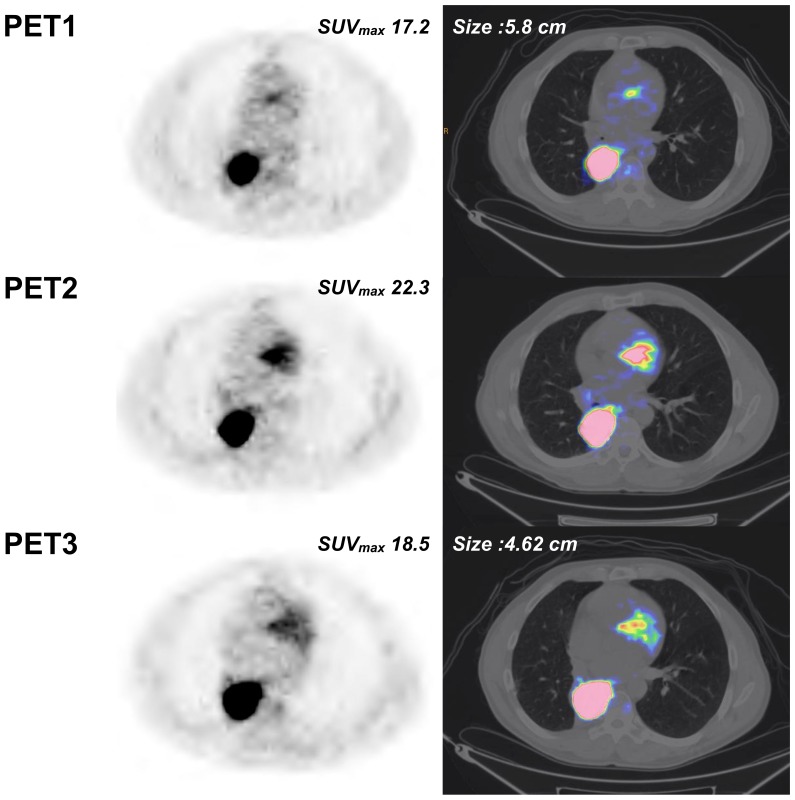
Example of a patient with discordant PET2 and conventional imaging. Patient with right upper lobe NSCLC associated with subcarinal lymphadenopathy and ipsilateral lung metastasis (patient #9). Sum of the SUVmax of the most hypermetabolic lesions (2 lung lesions, 1 mediastinal lymph node) were 25.2, 29.3 (+16.3%) and 23.8 (−5.4%) for PET1, PET2 (% versus PET1) and PET3 (% versus PET1), respectively. Based on a SUVmax cut-off value of −21.6, the patient was classified as mP on PET2, in contrast with RECIST evaluation on CT scan (performed 71 days after starting erlotinib). This patient was subsequently reclassified as mNP on PET3 in accordance with RECIST evaluation with a 5.4% decrease of SUVmax (cut-off: 18.5%).

On ROC analysis, the AUC for prediction of non-progressive disease by PET2 was 0.86 (95% CI, 0.62 to 1.1; P = 0.04), corresponding to a maximum specificity of 0.80 and sensitivity of 0.86 for non-progressive disease at a cut-off of 21.6% reduction in SUVmax (Figure 1) and a positive predictive value (PPV) of 0.86, a negative predictive value (NPV) of 0.80, an accuracy of 0.83 and a maximum Youden index of 0.65. The use of this SUVmax cut-off value correctly classified 11/12 patients (7 with true progressive disease (Figures 2 and 3); 4 with true non-progressive disease (Figures 4 and 5); 1 with false progressive disease (Figure 6). Non-progression after 2 months of treatment was significantly more frequent in patients with an early decrease in SUVmax of 21.6% or more (P = 0.01, Fisher's exact test). The only misclassified patient (patient #9, false progressive disease on PET2 versus PET1) displayed a 16.4% increase of SUVmax, but metabolic progression was not confirmed on PET3, with a 5.4% decrease of SUVmax compared to PET1. Similar results were observed for SUVpeak, as non-progressive disease after 2 months of treatment was significantly more frequent in patients with a decrease in SUVpeak of at least 17.6% on PET2 (P = 0.01, Fisher's exact test). Similar results were also obtained in terms of AUC, sensitivity, specificity, PPV, NPV, and accuracy and with the same classification of patients (7 with true progressive disease; 4 with true non-progressive disease; 1 with false progressive disease).

In 9/10 patients, semi-quantitative analysis on PET3 revealed response information concordant with PET2 studies. ROC analyses were also performed for SUV changes between PET1 and PET3. For SUVmax, sensitivity, specificity, PPV, NPV and accuracy were 0.8, 1, 0.83, 1 and 0.9, respectively, for an −18.5% cut-off value and an AUC of 0.76 (95% CI; 0.44 to 1.08; P = 0.17). For SUVpeak, sensitivity, specificity, PPV, NPV and accuracy were 1, 0.8, 1, 0.83 and 0.9, respectively, for a −3.9% cut-off value with an AUC of 0.8 (95% CI; 0.5108 to 1.089; P = 0.12). Patients were classified identically with SUVmax and SUVpeak (4 with true progressive disease, 5 with true non-progressive disease and one with false non-progressive disease). Due to the appearance of new lesions on PET, the patient #7 who was falsely classified as NP by semi-quantitative analysis of PET was correctly reclassified as P. Finally, PET3 correctly classified all 10 patients (5 in group P; 5 in group NP) in whom a third [^18^F]FDG-PET was performed, when compared with RECIST evaluation (P = .0079, Fisher's exact test).

### Patient outcome

**Figure 7:**
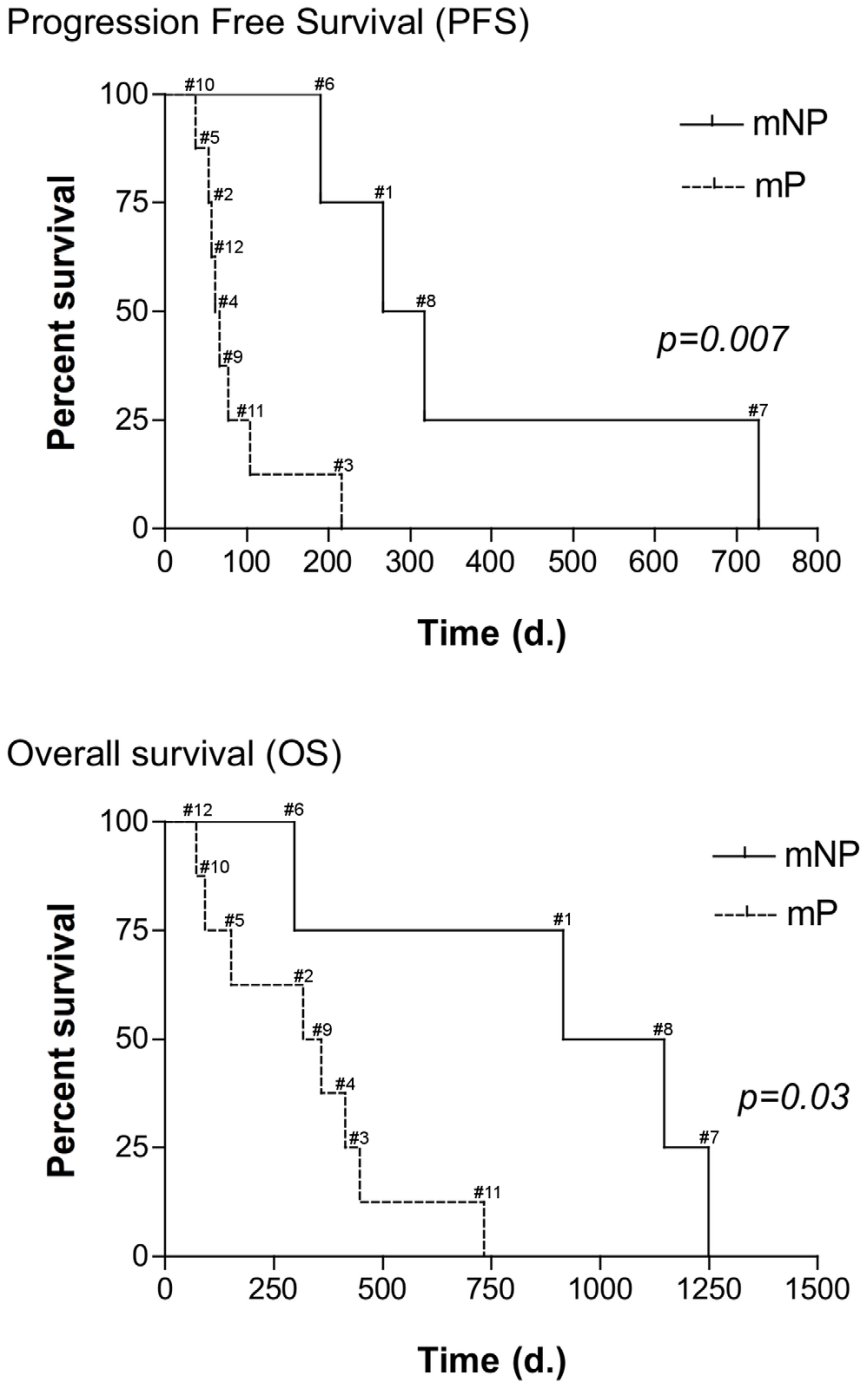
Kaplan-Meier estimates of PFS and OS. No statistically significant difference (P = 0.007) in PFS was observed between metabolic non-progressive (mNP) patients (median PFS, 292 days ; range, 190–727) and metabolic (mP) progressive patients (median PFS, 64 days ; range: 37–216). Improved PFS in non-progressive patients was associated with prolonged OS (mNP; n = 4; median OS: 1031 days ; 296 to 1249 days versus mP; n = 8 ; 337, 5 days ; 71 to 734 days) (HR, 0.34; 95% CI, 0.06 to 0.84; P = 0.03).

PFS and OS were 91 and 338 days, respectively. Using the SUVmax or SUVpeak cut-off defined by ROC analyses on PET2, patients were classified into 2 groups: metabolic progressive (n = 8; mP) or metabolic non-progressive (n = 4; mNP). mNP patients showed prolonged PFS (n = 4; median survival 292 days) compared to mP patients (n = 8; median 64 days) (HR, 0.27; 95% CI, 0.04 to 0.59; P = 0.007; Figure 7). Improved PFS observed in mNP patients was followed by prolonged OS (1031 days versus 1249 days; HR, 0.34; 95% CI, 0.06 to 0.84; P = 0.03; Figure 7). The first patient with EGFR mutation showed a PFS and OS of only 190 days and 296 days, respectively, due to erlotinib toxicity (grade IV neurotoxicity) resulting in early discontinuation of treatment. The second patient with EGFR mutation achieved the longest PFS and OS (727 and 1249 days, respectively).

## Discussion

Despite the widespread use of [^18^F]FDG-PET/CT in NSCLC staging, a large-scale study recently failed to confirm an overall survival gain in NSCLC patients.[17] This result highlights the value of [^18^F]FDG-PET/CT in unmet clinical needs, such as prediction of residual NSCLC after surgery[18], neoadjuvant therapy[19] or antineoplastic therapy.[20] Prediction of response to antineoplastic therapies would appear to be particularly adapted to targeted therapies that do not induce rapid tumor shrinkage. NSCLC preclinical models have validated this hypothesis with both gefitinib[21] and erlotinib.[22] This original method could compensate for the weakness of RECIST criteria and has led to the proposal of evaluation of new criteria by addition metabolic evaluation by FDG-PET to CT scan.[23] The value of PET in evaluation of response to new targeted therapies emerged in the early 2000 s with the first reports on the efficacy of imatinib mesylate in Gastro Intestinal Stromal Tumor (GIST). Subsequently, many studies have confirmed that PET is able to identify very early (i.e. only 24 hours after initiation of treatment) a decrease in glucose metabolism, which is correlated with overall survival and progression-free survival of patients with GIST.[24], [25]

In the present exploratory study, a decrease in SUVmax of at least 21.6% soon after starting therapy (9±3 days) was able to discriminate progressive from non-progressive patients and was associated with improved PFS and OS. This result confirms the results of Mileshkin et al., who showed, in a series of 51 patients receiving second- or third-line treatment with erlotinib, that an early (14 days) [^18^F]FDG-PET partial metabolic response was associated with improved PFS and OS, even in the absence of subsequent RECIST response.[26]

Evaluation of response by [^18^F]FDG-PET can be performed semi-quantitatively, for instance by establishing a SUV cut-off to discriminate metabolic progressive patients from non-metabolic progressive patients. This patient classification (mP/mNP) seems to be more appropriate to assess response to cytostatic therapy that is designed to stabilize disease, rather than achieve complete response. The main difficulty of this approach is the overlap of SUV changes between mP and mNP patients. Furthermore, different cut-off variations can be expected depending on the types of SUV measured, the types of drugs used and the types of tumors, which increase the difficulty of establishing a reliable SUV cut-off. However, despite the absence of consensus on the most appropriate cut-off value, it is generally admitted that the rationale for metabolic response or non-progression of tumor is decreased [^18^F]FDG tumor uptake or at least stability of tumor uptake over time, respectively.

Another limitation of semi-quantitative analysis of FDG-PET is that it does not take into account the development of new lesions. However, PET detection of new lesions early in the course of therapy has been reported to be a strong, independent predictive factor of OS in NSCLC patients treated by EGFR inhibitor.[27] Our findings are consistent with this observation, as new lesions occurred in 2/8 patients correctly classified as progressive on PET2 and in 4/5 patients correctly classified as progressive on PET3. One patient (patient #7) was reclassified as mP on PET3 due to the appearance of a new lesion, despite a decrease of SUVmax to below the cut-off value.

As in our study, previous studies failed to demonstrate any difference between SUVmax and SUVpeak.[22], [28] However, SUVmax remains the standard for semi-quantitative [^18^F]FDG-PET assessment, probably because is a parameter that can be reliably reproduced by independent operators. It is noteworthy that, in our study, no significant difference in mean SUV values was observed between PET1, PET2 and PET3, which can be explained by the nature of the cytostatic therapy.

11/12 patients were correctly classified (P versus NP) by PET2 and 10/10 were correctly classified by PET3 by applying the SUV cut-off determined by ROC analysis. In 9/10 patients, PET3 revealed response information concordant with PET2. The only patient with discordant [^18^F]FDG-PET findings was classified by SUV analysis as progressive on PET2 and non-progressive on PET3. Blood glucose, injected dose or uptake time were normal and/or not significantly different between PET2 and PET3 (1.16 and 1.4 g/l; 261 and 262 MBq; 60 and 75 min, respectively) excluding any to methodology-related error. A flare-up phenomenon could be proposed, as described on several occasions on [^18^F]FDG-PET during cytotoxic treatments for squamous cell carcinoma, in prostate cancer patients with bone metastases[29]–[33] and particularly NSCLC patients treated with erlotinib presenting an osteoblastic bone flare-up response mimicking disease progression.[34] Benz et al also described a case of flare-up on early PET in a NSCLC patient treated by erlotinib.[27] Another explanation is that the P/NP classification probably increases mismatches of response assessments, related to a discordant outcome of patients with stable disease.[27]

Our results suggest that therapeutic efficacy, PFS and OS of erlotinib therapy can be predicted 2 weeks after starting erlotinib. These data are consistent with the data of a retrospective study recently published by Kobe et al.[26], [35] At the present time, anticancer therapy is currently monitored in the context of hormone-sensitive cancers by regular assay of tumor markers (such as prostate-specific antigen in prostate cancer). The efficacy of hormonal therapy is reflected by a decrease in blood levels of the marker. When the marker remains elevated, hormonal therapy is considered to be ineffective and is therefore stopped. Repeated PET imaging can be considered to be a promising approach to evaluate cancer therapy such as targeted therapies that do not induce tumor shrinkage. This new approach appears to be supported by the results of recent clinical trials. The ‘Tarceva Versus Docetaxel or Pemetrexed for Second Line Chemotherapy of Advanced Stage NSCLC’ (TITAN) trial failed to demonstrate an improvement in OS with erlotinib compared to chemotherapy in unselected NSCLC patients receiving second-line treatment (HR = 0.96; 95% CI, 0.78–1.19; p = 0.73).[36] In a similar group of NSCLC patients, the results of the TAILOR trial indicated a highly significant increase of PFS in favor of docetaxel (HR = 0.71; 95% CI, 0.53–0.95; p = 0.02) versus erlotinib.[37] We consider that evaluation of the metabolic response to erlotinib could provide useful information to rapidly identify patients in whom erlotinib therapy is ineffective, especially in EGFR patients without EGFR-activating mutations or unknown status. [^18^F]FDG-PET could also become a theranostic tool for clinicians. By stopping ineffective therapy earlier, physicians can rapidly propose other drugs to a larger proportion of patients with better performance status.

This approach could increase the number of patients included in early trials and accelerate drug development. However, no medico-economic study has been conducted to determine whether the additional costs induced by [^18^F]FDG-PET are compensated by the decreased costs of drug (erlotinib) and medical care induced by side effects. Our study highlights the need for more prospective and randomized studies to evaluate the theranostic use of [^18^F]FDG-PET for management of erlotinib therapy in NSCLC, including medico-economic considerations.

## Conclusion

[^18^F]FDG-PET performed within two weeks of starting erlotinib therapy (9±3 days) appears to be able to predict morphologic response at 2 months according to RECIST criteria. [^18^]FDG-PET may be clinically useful for early evaluation of targeted therapies as a theranostic tool.

## References

[1] Ferlay J, Parkin DM, Steliarova-Foucher E (2010) Estimates of cancer incidence and mortality in Europe in 2008. Eur J Cancer 46: 765–781. 20116997 10.1016/j.ejca.2009.12.014

[2] Jemal A, Bray F, Center MM, Ferlay J, Ward E, et al (2011) Global cancer statistics. CA Cancer J Clin 61: 69–90. 21296855 10.3322/caac.20107

[3] Chemotherapy in non-small cell lung cancer: a meta-analysis using updated data on individual patients from 52 randomised clinical trials. Non-small Cell Lung Cancer Collaborative Group. BMJ 311: 899–909. PMC2550915 7580546

[4] Schiller JH, Harrington D, Belani CP, Langer C, Sandler A, et al (2002) Comparison of four chemotherapy regimens for advanced non-small-cell lung cancer. N Engl J Med 346: 92–98. 11784875 10.1056/NEJMoa011954

[5] Lynch TJ, Bell DW, Sordella R, Gurubhagavatula S, Okimoto RA, et al (2004) Activating mutations in the epidermal growth factor receptor underlying responsiveness of non-small-cell lung cancer to gefitinib. N Engl J Med 350: 2129–2139. 15118073 10.1056/NEJMoa040938

[6] Mok TS, Wu YL, Thongprasert S, Yang CH, Chu DT, et al (2009) Gefitinib or carboplatin-paclitaxel in pulmonary adenocarcinoma. N Engl J Med 361: 947–957. 19692680 10.1056/NEJMoa0810699

[7] Rosell R, Carcereny E, Gervais R, Vergnenegre A, Massuti B, et al (2012) Erlotinib versus standard chemotherapy as first-line treatment for European patients with advanced EGFR mutation-positive non-small-cell lung cancer (EURTAC): a multicentre, open-label, randomised phase 3 trial. Lancet Oncol 13: 239–246. 22285168 10.1016/S1470-2045(11)70393-X

[8] Shepherd FA, Rodrigues Pereira J, Ciuleanu T, Tan EH, Hirsh V, et al (2005) Erlotinib in previously treated non-small-cell lung cancer. N Engl J Med 353: 123–132. 16014882 10.1056/NEJMoa050753

[9] Cappuzzo F, Ciuleanu T, Stelmakh L, Cicenas S, Szczesna A, et al (2010) Erlotinib as maintenance treatment in advanced non-small-cell lung cancer: a multicentre, randomised, placebo-controlled phase 3 study. Lancet Oncol 11: 521–529. 20493771 10.1016/S1470-2045(10)70112-1

[10] Miller AB, Hoogstraten B, Staquet M, Winkler A (1981) Reporting results of cancer treatment. Cancer 47: 207–214. 7459811 10.1002/1097-0142(19810101)47:1<207::aid-cncr2820470134>3.0.co;2-6

[11] Therasse P, Arbuck SG, Eisenhauer EA, Wanders J, Kaplan RS, et al (2000) New guidelines to evaluate the response to treatment in solid tumors. European Organization for Research and Treatment of Cancer, National Cancer Institute of the United States, National Cancer Institute of Canada. J Natl Cancer Inst 92: 205–216. 10655437 10.1093/jnci/92.3.205

[12] Eisenhauer EA, Therasse P, Bogaerts J, Schwartz LH, Sargent D, et al (2009) New response evaluation criteria in solid tumours: revised RECIST guideline (version 1.1). Eur J Cancer 45: 228–247. 19097774 10.1016/j.ejca.2008.10.026

[13] Fischer BM, Mortensen J, Hojgaard L (2001) Positron emission tomography in the diagnosis and staging of lung cancer: a systematic, quantitative review. Lancet Oncol 2: 659–666. 11902536 10.1016/S1470-2045(01)00555-1

[14] Lardinois D, Weder W, Hany TF, Kamel EM, Korom S, et al (2003) Staging of non-small-cell lung cancer with integrated positron-emission tomography and computed tomography. N Engl J Med 348: 2500–2507. 12815135 10.1056/NEJMoa022136

[15] Vansteenkiste JF, Stroobants SG, Dupont PJ, De Leyn PR, Verbeken EK, et al (1999) Prognostic importance of the standardized uptake value on (18)F-fluoro-2-deoxy-glucose-positron emission tomography scan in non-small-cell lung cancer: An analysis of 125 cases. Leuven Lung Cancer Group. J Clin Oncol 17: 3201–3206. 10506619 10.1200/JCO.1999.17.10.3201

[16] Berghmans T, Dusart M, Paesmans M, Hossein-Foucher C, Buvat I, et al (2008) Primary tumor standardized uptake value (SUVmax) measured on fluorodeoxyglucose positron emission tomography (FDG-PET) is of prognostic value for survival in non-small cell lung cancer (NSCLC): a systematic review and meta-analysis (MA) by the European Lung Cancer Working Party for the IASLC Lung Cancer Staging Project. J Thorac Oncol 3: 6–12. 18166834 10.1097/JTO.0b013e31815e6d6b

[17] Dinan MA, Curtis LH, Carpenter WR, Biddle AK, Abernethy AP, et al (2012) Stage migration, selection bias, and survival associated with the adoption of positron emission tomography among medicare beneficiaries with non-small-cell lung cancer, 1998-2003. J Clin Oncol 30: 2725–2730. 22753917 10.1200/JCO.2011.40.4392

[18] Velazquez ER, Aerts HJ, Oberije C, De Ruysscher D, Lambin P (2010) Prediction of residual metabolic activity after treatment in NSCLC patients. Acta Oncol 49: 1033–1039. 20831492 10.3109/0284186X.2010.498441

[19] Aukema TS, Kappers I, Olmos RA, Codrington HE, van Tinteren H, et al (2010) Is 18F-FDG PET/CT useful for the early prediction of histopathologic response to neoadjuvant erlotinib in patients with non-small cell lung cancer? J Nucl Med 51: 1344–1348. 20720059 10.2967/jnumed.110.076224

[20] planned T, Scheffler M, Nogova L, Kobe C, Engel-Riedel W, et al (2011) Early prediction of nonprogression in advanced non-small-cell lung cancer treated with erlotinib by using [(18)F]fluorodeoxyglucose and [(18)F]fluorothymidine positron emission tomography. J Clin Oncol 29: 1701–1708. 21422426 10.1200/JCO.2010.32.4939

[21] Su H, Bodenstein C, Dumont RA, Seimbille Y, Dubinett S, et al (2006) Monitoring tumor glucose utilization by positron emission tomography for the prediction of treatment response to epidermal growth factor receptor kinase inhibitors. Clin Cancer Res 12: 5659–5667. 17020967 10.1158/1078-0432.CCR-06-0368

[22] Ullrich RT, Zander T, Neumaier B, Koker M, Shimamura T, et al (2008) Early detection of erlotinib treatment response in NSCLC by 3′-deoxy-3′-[F]-fluoro-L-thymidine ([F]FLT) positron emission tomography (PET). PLoS One 3: e3908. 19079597 10.1371/journal.pone.0003908 PMC2592703

[23] Wahl RL, Jacene H, Kasamon Y, Lodge MA (2009) From RECIST to PERCIST: Evolving Considerations for PET response criteria in solid tumors. J Nucl Med 50 Suppl 1122S–150S. 19403881 10.2967/jnumed.108.057307 PMC2755245

[24] Stroobants S, Goeminne J, Seegers M, Dimitrijevic S, Dupont P, et al (2003) 18FDG-Positron emission tomography for the early prediction of response in advanced soft tissue sarcoma treated with imatinib mesylate (Glivec). Eur J Cancer 39: 2012–2020. 12957455 10.1016/s0959-8049(03)00073-x

[25] Van den Abbeele AD (2008) The lessons of GIST–PET and PET/CT: a new paradigm for imaging. Oncologist 13 Suppl 28–13. 18434632 10.1634/theoncologist.13-S2-8

[26] Mileshkin L, Hicks RJ, Hughes BG, Mitchell PL, Charu V, et al (2011) Changes in 18F-fluorodeoxyglucose and 18F-fluorodeoxythymidine positron emission tomography imaging in patients with non-small cell lung cancer treated with erlotinib. Clin Cancer Res 17: 3304–3315. 21364032 10.1158/1078-0432.CCR-10-2763

[27] Benz MR, Herrmann K, Walter F, Garon EB, Reckamp KL, et al (2011) (18)F-FDG PET/CT for monitoring treatment responses to the epidermal growth factor receptor inhibitor erlotinib. J Nucl Med 52: 1684–1689. 22045706 10.2967/jnumed.111.095257 PMC5021512

[28] Kahraman D, Scheffler M, Zander T, Nogova L, Lammertsma AA, et al (2011) Quantitative analysis of response to treatment with erlotinib in advanced non-small cell lung cancer using 18F-FDG and 3′-deoxy-3′-18F-fluorothymidine PET. J Nucl Med 52: 1871–1877. 22065872 10.2967/jnumed.111.094458

[29] Bjurberg M, Henriksson E, Brun E, Ekblad L, Ohlsson T, et al (2009) Early changes in 2-deoxy-2-[18F]fluoro-D-glucose metabolism in squamous-cell carcinoma during chemotherapy in vivo and in vitro. Cancer Biother Radiopharm 24: 327–332. 19538055 10.1089/cbr.2008.0556

[30] Messiou C, Cook G, Reid AH, Attard G, Dearnaley D, et al (2011) The CT flare response of metastatic bone disease in prostate cancer. Acta Radiol 52: 557–561. 21498309 10.1258/ar.2011.100342

[31] Krupitskaya Y, Eslamy HK, Nguyen DD, Kumar A, Wakelee HA (2009) Osteoblastic bone flare on F18-FDG PET in non-small cell lung cancer (NSCLC) patients receiving bevacizumab in addition to standard chemotherapy. J Thorac Oncol 4: 429–431. 19247091 10.1097/JTO.0b013e3181989e12

[32] Biersack HJ, Bender H, Palmedo H (2004) FDG-PET in monitoring therapy of breast cancer. Eur J Nucl Med Mol Imaging 31 Suppl 1S112–117. 15112111 10.1007/s00259-004-1533-x

[33] Mortimer JE, Dehdashti F, Siegel BA, Trinkaus K, Katzenellenbogen JA, et al (2001) Metabolic flare: indicator of hormone responsiveness in advanced breast cancer. J Clin Oncol 19: 2797–2803. 11387350 10.1200/JCO.2001.19.11.2797

[34] Lind JS, Postmus PE, Smit EF (2010) Osteoblastic bone lesions developing during treatment with erlotinib indicate major response in patients with non-small cell lung cancer: a brief report. J Thorac Oncol 5: 554–557. 20357621 10.1097/JTO.0b013e3181d3e47e

[35] Kobe C, Scheffler M, Holstein A, Zander T, Nogova L, et al (2012) Predictive value of early and late residual 18F-fluorodeoxyglucose and 18F-fluorothymidine uptake using different SUV measurements in patients with non-small-cell lung cancer treated with erlotinib. Eur J Nucl Med Mol Imaging 39: 1117–1127. 22526960 10.1007/s00259-012-2118-8

[36] Ciuleanu T, Stelmakh L, Cicenas S, Miliauskas S, Grigorescu AC, et al (2012) Efficacy and safety of erlotinib versus chemotherapy in second-line treatment of patients with advanced, non-small-cell lung cancer with poor prognosis (TITAN): a randomised multicentre, open-label, phase 3 study. Lancet Oncol 13: 300–308. 22277837 10.1016/S1470-2045(11)70385-0

[37] Garassino MC, Martelli O, Broggini M, Farina G, Veronese S, et al (2013) Erlotinib versus docetaxel as second-line treatment of patients with advanced non-small-cell lung cancer and wild-type EGFR tumours (TAILOR): a randomised controlled trial. Lancet Oncol 14: 981–988. 23883922 10.1016/S1470-2045(13)70310-3

